# Preoperative Prediction of Lymph Node Metastasis of Pancreatic Ductal Adenocarcinoma Based on a Radiomics Nomogram of Dual-Parametric MRI Imaging

**DOI:** 10.3389/fonc.2022.927077

**Published:** 2022-07-06

**Authors:** Lin Shi, Ling Wang, Cuiyun Wu, Yuguo Wei, Yang Zhang, Junfa Chen

**Affiliations:** ^1^ Cancer Center, Department of Radiology, Zhejiang Provincial People’s Hospital (Affiliated People’s Hospital, Hangzhou Medical College), Hangzhou, China; ^2^ Precision Health Institution, General Electric Healthcare, Hangzhou, China

**Keywords:** pancreatic ductal adenocarcinoma, magnetic resonance imaging, radiomics, lymph node metastasis, nomogram

## Abstract

**Purpose:**

This study aims to uncover and validate an MRI-based radiomics nomogram for detecting lymph node metastasis (LNM) in pancreatic ductal adenocarcinoma (PDAC) patients prior to surgery.

**Materials and Methods:**

We retrospectively collected 141 patients with pathologically confirmed PDAC who underwent preoperative T2-weighted imaging (T2WI) and portal venous phase (PVP) contrast-enhanced T1-weighted imaging (T1WI) scans between January 2017 and December 2021. The patients were randomly divided into training (n = 98) and validation (n = 43) cohorts at a ratio of 7:3. For each sequence, 1037 radiomics features were extracted and analyzed. After applying the gradient-boosting decision tree (GBDT), the key MRI radiomics features were selected. Three radiomics scores (rad-score 1 for PVP, rad-score 2 for T2WI, and rad-score 3 for T2WI combined with PVP) were calculated. Rad-score 3 and clinical independent risk factors were combined to construct a nomogram for the prediction of LNM of PDAC by multivariable logistic regression analysis. The predictive performances of the rad-scores and the nomogram were assessed by the area under the operating characteristic curve (AUC), and the clinical utility of the radiomics nomogram was assessed by decision curve analysis (DCA).

**Results:**

Six radiomics features of T2WI, eight radiomics features of PVP and ten radiomics features of T2WI combined with PVP were found to be associated with LNM. Multivariate logistic regression analysis showed that rad-score 3 and MRI-reported LN status were independent predictors. In the training and validation cohorts, the AUCs of rad-score 1, rad-score 2 and rad-score 3 were 0.769 and 0.751, 0.807 and 0.784, and 0.834 and 0.807, respectively. The predictive value of rad-score 3 was similar to that of rad-score 1 and rad-score 2 in both the training and validation cohorts (P > 0.05). The radiomics nomogram constructed by rad-score 3 and MRI-reported LN status showed encouraging clinical benefit, with an AUC of 0.845 for the training cohort and 0.816 for the validation cohort.

**Conclusions:**

The radiomics nomogram derived from the rad-score based on MRI features and MRI-reported lymph status showed outstanding performance for the preoperative prediction of LNM of PDAC.

Pancreatic cancer, as a highly malignant gastrointestinal tumor, has a five-year mortality rate close to its morbidity rate (1, 2). Pancreatic ductal adenocarcinoma (PDAC) is the predominant histological subtype, accounting for 85% of all pancreatic cancer cases (3). Schwarz et al. (4) conducted a retrospective analysis of 2787 patients who underwent surgical resection (SR) for pancreatic cancer in the United States and found that 54% of patients had lymph node metastasis (LNM), suggesting that LNM is a potential key to assess the state of the disease, as it influences the formulation of surgical procedures and patient prognosis (5). Different preoperative noninvasive examinations, including computed tomography (CT), magnetic resonance imaging (MRI) and positron emission tomography (PET), are commonly used to identify LNM of pancreatic cancer (6–11). Unfortunately, all of these technologies are still inadequate for assessing LNM status because enlarged lymph nodes are often caused by nonspecific inflammation (12). In addition, although endoscopic ultrasonography (EUS) has high sensitivity for the diagnosis of pancreatic primary lesions and LNM and sufficient histological information can be obtained from a small sample of tissue, it is an invasive method (13, 14). Its use is also limited by several other factors, such as the focal size and surrounding anatomical environment, yielding an accuracy of 41-86% for lymph node staging of pancreatic adenocarcinoma (14). Recent studies have shown that Ki-67 and serum MMP7 have the potential to predict LNM, but their sensitivities remain insufficient (15, 16).

Radiomics approaches allow for the quantitative analysis of images and can reflect heterogeneity in the region of interest (ROI), providing more information through feature analysis than can be recognized by the naked eye, making it helpful for clarifying the nature of lesions (17). T2-weighted imaging (T2WI) and the dynamic enhanced portal venous phase (PVP) of MRI can better depict the biological characteristics of pancreatic lesions and have therefore been applied to radiomics studies of pancreatic cancer (18–20), including for differential diagnosis, prognosis evaluations, and treatment response predictions. Although studies have shown that radiomics can be used for the preoperative prediction of LNM of malignant tumors (21–24), few radiomics studies based on MRI image texture analysis have been conducted for the preoperative prediction of LNM of PDAC. Therefore, this study aimed to explore whether the use of T2WI and PVP features was feasible for predicting LNM of PDAC. We sought to develop and validate a radiomics nomogram as a noninvasive and feasible approach for the preoperative detection of LNM in PDAC patients.

## Materials and Methods

### Patients

**Figure 1:**
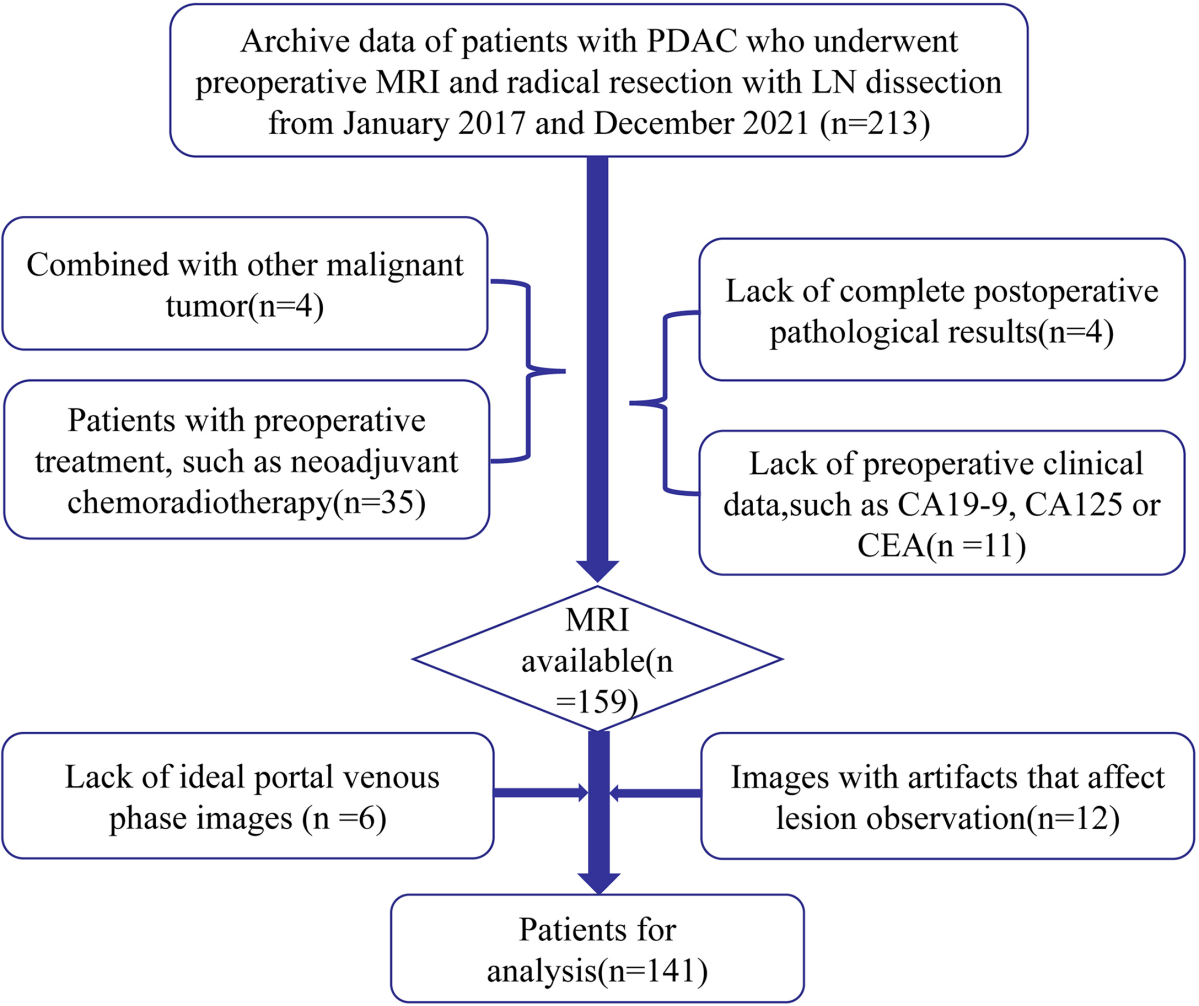
Patient selection flowchart.

This study was approved by the Ethics Committee of the Zhejiang Provincial People’s Hospital, Affiliated People’s Hospital of Hangzhou Medical College. The requirement for informed consent was waived due to the retrospective nature of this study. Clinical and MRI databases of patients were retrospectively reviewed to identify candidate patients who were treated between January 2017 and December 2021. The inclusion criteria were as follows: (1) patients who received radical resection and regional lymph node dissection for PDAC diagnosed by postoperative pathology and (2) patients with PDAC who underwent dynamic enhancement MRI scanning within two weeks before SR. The exclusion criteria were as follows: (1) images with artifacts that affected lesion observation; (2) patients who received any treatment for PDAC before SR, such as neoadjuvant chemoradiotherapy; and (3) patients with PDAC and other malignant tumors. Among the 141 patients who met these criteria, 58 were diagnosed with LNM. All patients were randomly divided into training (n = 98) and validation (n = 43) cohorts at a ratio of 7:3. Clinical data of the patients were collected, including sex, age, primary tumor site, the maximum diameter of the tumor, MRI tumor stage (mTs), MRI-reported lymph node status, and the levels of carbohydrate antigen 19-9 (CA19-9), carbohydrate antigen 125 (CA125) and carcinoma embryonic antigen (CEA). A positive lymph node on MRI was defined as a nodule at least 10 mm in the short-axis diameter or a nodule with a round shape, heterogeneous enhancement and low ADC value (25). The patient selection flowchart is shown in **Figure 1**.

### MRI Protocol

MRI was performed with a 3.0 T Discovery MR 750 scanner (GE Healthcare, Waukesha, WI, United States). (1) The following parameters were used for fat-suppressed fast spin-echo T2-weighted imaging (T2WI): repetition time (TR)/echo time (TE), 12000/72 ms; matrix size, 320 × 320; field of view (FOV), 360 × 360 mm^2^; slice thickness, 3 mm; spacing between slices, 0.6 mm; number of excitation (NEX), 2; and bandwidth, 83.3 kHz. (2) Gd-diethylenetriamine pentaacetic acid (Gd-DTPA) was injected at a dose of 0.1 mmol/kg through the median cubital vein at an injection rate of 2.0 mL/s, followed by 15 ml of saline at the same flow rate. A fat-suppressed T1-weighted three-dimensional (3D) gradient-recalled-echo sequence was used to collect dynamic enhanced images with the following parameters: TR/TE, 4.1/1.2 ms; matrix size, 260 × 240; FOV, 360 × 360 mm^2^; slice thickness, 3 mm; spacing between slices, 0 mm; NEX, 1; and bandwidth, 142.8 kHz. The late arterial phase (LAP), portal venous phase (PVP), and delayed phase (DP) were acquired at 25 seconds, 45 seconds, and 80 seconds. Other scanning sequence conditions not used for radiomics are not listed in this study.

### Tumor Segmentation and Feature Extraction

**Figure 2:**
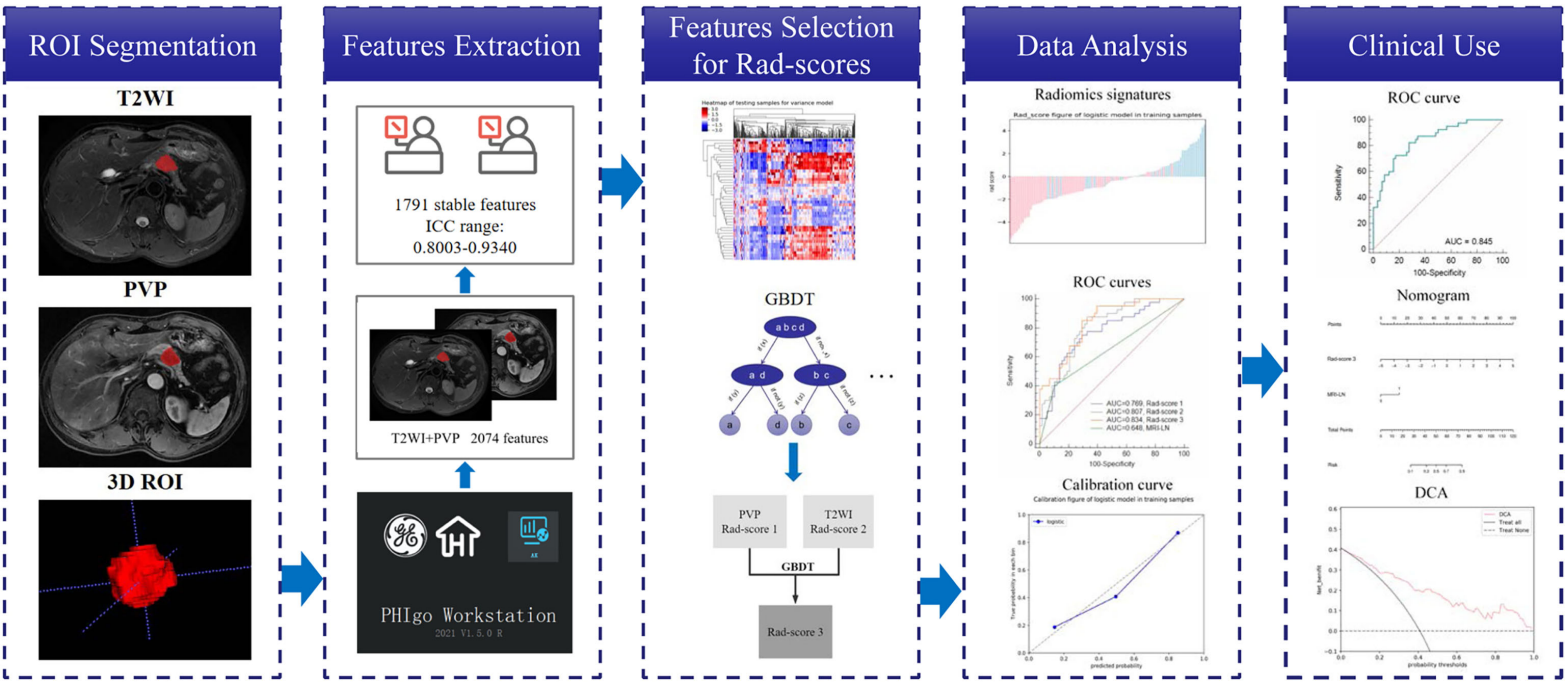
Radiomics and model construction workflow.

Using ITK-SNAP software (26) (**Figure 2**), segmentation of the regions of interest (ROIs) was performed by two independent radiologists with 5 and 15 years of experience in abdominal radiology, named reader 1 and reader 2, respectively. With reference to diffusion weighted imaging (DWI) and dynamic enhanced images, 3D ROIs based on T2WI and PVP were drawn manually. Features of ROIs were extracted by PHIgo software (GE Healthcare, V1.2.0, China), which is based on pyradiomics, and complies with the image biomarker standardization initiative (IBSI) (27). Prior to this, all images underwent standardized preprocessing, including image resampling at the same resolution (1*1*1 mm^3^) and dividing the gray level into grades 1-10. A total of 1037 features were obtained, including first-order features, shape features, gray level cooccurrence matrix (GLCM) features, gray level size zone matrix (GLSZM) features, gray level run length matrix (GLRLM) features, neighboring gray tone difference matrix (NGTDM) features and gray level dependence matrix (GLDM) features. The stability and reliability were evaluated using intraclass correlation coefficients (ICCs) by comparing 30 random patients’ ROIs drawn by reader 1 and reader 2. Features with ICC values > 0.8 were interpreted as almost perfect and recorded.

### Dimensionality Reduction and Radiomics Score Calculation

Dimensionality reduction for T2WI and PVP was performed using analysis of variance and the Mann–Whitney U test, Spearman’s correlation, and gradient boosting decision tree (GBDT) in sequence. Combining the selected features from T2WI and PVP, GBDT was again used to select significant features. Radiomics scores (rad-score 1, rad-score 2 and rad-score 3) were calculated based on the remaining features from T2WI, PVP and T2WI combined with PVP by multivariate logistic regression.

### Radiomics Nomogram Development and Evaluation

Univariate logistic regression analysis and multivariate logistic regression analysis were performed with the clinical characteristics and rad-score 3 to identify potential and independent predictors of LNM, respectively (28). Finally, a radiomics nomogram was constructed with the identified predictors of LNM. The Hosmer–Lemeshow test and calibration curves were used to assess the goodness-of-fit and calibration of the nomogram (29). The predictive performances of the clinical model, three rad-scores, and the nomogram for LNM were evaluated by receiver operator characteristic (ROC) curve analysis, and the areas under the curve (AUCs) were calculated. Decision curve analysis (DCA) was performed to determine the clinical efficiency of the nomogram.

### Statistical Analysis

The data were analyzed by SPSS 22.0 (IBM Corporation), MedCalc (Version 14.10.20) and Microsoft R Open (version 3.3.1) software. Univariate analysis was used to assess the correlations between the clinical characteristics and LNM, with the chi-square test used for categorical variables and the two-sample t test used for continuous variables. Normality was assessed by the Kolmogorov–Smirnov test. The variables that followed a normal distribution are expressed as the mean ± standard deviation, and nonnormally distributed variables are expressed as the median (interquartile range). The De-Long test was used for statistical comparison of the AUCs of the models. Calibration plots and DCA were performed using the “rms” and “dca” packages in R (Microsoft R Open; version 3.3.1), respectively. All statistical tests were two-tailed, and significance was set at *P*<0.05.

## Results

### Clinical Characteristics

There were no significant differences in any of the clinical characteristics between the training and validation cohorts (**Table 1**), and the LN positivity rate was not significantly different between the two cohorts (40.8% (40/98) vs. 41.9% (18/43), respectively; P=0.908). The only significant difference among the clinical characteristics was in the MRI-reported LNM status between patients with LNM and those with non-lymph node metastasis (nLNM) in both the training and validation cohorts (*P*<0.05) (**Table 1**).

**Table 1:** Patients’ clinical characteristics and rad-scores in the training and validation cohorts.

Characteristic	Training		*P*	Validation		*P*	*P*
	nLNM	LNM		nLNM	LNM		
Age, mean ± SD	64.9 ± 9.60	64.65 ± 9.55	0.873	65.84 ± 6.79	64.28 ± 9.11	0.523	0.833
Sex							
Female, n (%)	19 (51.4%)	39 (63.9%)	0.219	9 (69.2%)	16 (53.3%)	0.332	0.390
Male, n (%)	18 (48.6%)	22 (36.1%)		4 (30.8%)	14 (46.7%)
Location							
Head/neck, n (%)	30 (52.6%)	28 (68.3%)	0.120	13 (56.5%)	12 (60%)	0.818	0.606
Body/tail, n (%)	27 (47.4%)	13 (31.7%)		10 (43.5%)	8 (40%)
Size (mm), median (IQR)	33.0 (25.0, 40.0)	33.0 (25.0, 41.5)	0.876	27.0 (20.0, 37.5)	34.0 (26.5, 41.3)	0.113	0.368
CA19-9 (U/ml), median (IQR)	116.80 (55.35, 366.85)	207.05 (75.58, 853.40)	0.120	86.3 (23.5, 214.75)	271.85 (63.03, 879.38)	0.028	0.478
CA125 (U/ml), median (IQR)	17.95 (10.70, 27.33)	16.90 (9.95, 31.9)	0.680	14.5 (7.2, 19.55)	22.25 (10.65, 45.83)	0.402	0.389
CEA (μg/ml), median (IQR)	3.35 (2.08, 5.95)	4.15 (2.28, 7.73)	0.278	3.30 (2.40, 5.35)	3.90 (2.58, 5.95)	0.076	0.846
mTs							
T1-2, n (%)	40 (59.7%)	18 (58.1%)	0.878	15 (60%)	10 (55.6%)	0.771	0.240
T3-4, n (%)	27 (40.3%)	13 (41.9%)		10 (40%)	8 (44.4%)
MRI-reported LN status							
Negative, n (%)	51 (89.5%)	24 (60.0%)	0.001	21 (84.0%)	4 (55.6%)	0.04	0.439
Positive, n (%)	6 (10.5%)	16 (40.0%)		10 (16.0%)	8 (44.4%)
Rad-score 1, mean ± SD	-0.921 ± 1.048	0.316 ± 1.424	0.000	-0.890 ± 0.946	0.035 ± 1.134	0.008	0.713
Rad-score 2, mean ± SD	-1.268 ± 1.626	0.611 ± 2.498	0.000	-1.364 ± 1.946	0.516 ± 2.498	0.006	0.841
Rad-score 3, mean ± SD	-1.351 ± 1.439	0.693 ± 1.579	0.000	-1.932 ± 1.573	-0.463 ± 1.654	0.005	0.722

SD, standard deviation; mTs, MRI tumor stage; IQR, interquartile range; LNM, lymph node metastasis; nLNM, non-lymph node metastasis.

### Radiomics Signature Development and Rad-Score Calculation

The ICC values for feature extraction between reader 1 and reader 2 ranged from 0.773 to 0.934, suggesting high agreement. A total of 1791 features were proven to have high consistency (ICCs: 0.8003-0.934). For the T2WI and PVP sequences, analysis of variance and the Mann-Whitney U test identified 480 and 478 important features, respectively. Following Spearman correlation analysis, the number of important features was reduced to 14 and 19. Finally, 6 and 8 features were ultimately identified by further GBDT dimensionality reduction. The corresponding rad-scores (1 and 2) were calculated based on the retained features included in the multivariable logistic regression. After merging the 14 features and using GBDT again, 10 features were obtained to calculate rad-score 3. Various features and coefficients of rad-score 3 are shown in **Table 2**, which are all wavelet features. The three rad-scores were significantly different between LNM and nLNM patients in both the training and validation cohorts (*P <*0.01), but there was no difference between the cohorts (**Table 1**).

**Table 2:** Radiomics features selected by GBDT.

Characteristic	β	OR	95% CI
PVP_wavelet-LLH_firstorder_Minimum	0.012	1.012	0.589,1.710
PVP_wavelet-LLH_glszm_SizeZoneNonUniformity	0.231	1.260	0.685,2.316
PVP_wavelet-LHH_glcm_MaximumProbability	0.790	2.203	1.132,4.289
PVP_wavelet-HHL_glcm_ClusterTendency	-0.519	0.595	0.343,1.034
PVP_wavelet-HHH_glszm_SmallAreaEmphasis	0.791	2.205	0.256,3.872
T2WI_wavelet-LLH_firstorder_Mean	0.971	2.640	1.276,5.462
T2WI_wavelet-HLH_glcm_ClusterShade	0.921	2.513	1.152,5.484
T2WI_wavelet-HHL_glcm_Correlation	0.473	1.604	0.903,2.852
T2WI_wavelet-HHH_gldm_DependenceNonUniformityNormalized	0.542	1.719	0.958,3.086
T2WI_wavelet-LLL_firstorder_Kurtosis	-0.641	0.527	0.273,1.016

OR, odds ratio; CI, confidence interval.

### Rad-Score Evaluation

**Figure 3:**
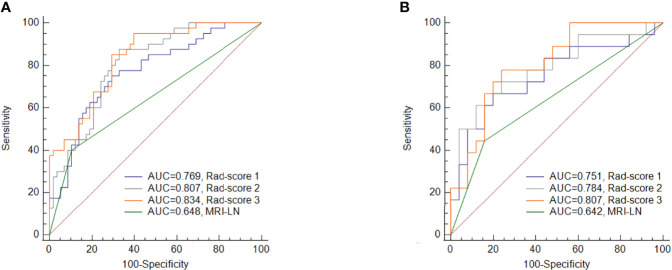
Comparisons of the ROC curves for MRI-reported LN status and the three rad-scores in the training cohort **(A)** and validation cohort **(B)**. MRI-LN, MRI-reported LN status.

The ROC curve demonstrates the predictive performance of the clinical model, rad-score 1, rad-score 2, and rad-score 3, as shown in **Figure 3**. In the training and validation cohorts, the AUCs of the clinical model, rad-score 1, rad-score 2 and rad-score 3 were 0.648, 0.642; 0.769, 0.751; 0.807, 0.784 and 0.834, 0.807, respectively. The thresholds for predicting LNM using rad-score 1, rad-score 2, and rad-score 3 were -0.441, -0.696, and -0.807, respectively, in the training cohort. Although the AUC value of rad-score 3 was the largest among the rad-scores in both the training and validation cohorts, the difference was not significant. Rad-score 2 and rad-score 3 had higher predictive efficacy than the clinical model in the training cohort (*P*<0.05), while rad-score 3 showed better performance than the clinical model in the validation cohort (*P <*0.05). Detailed results are shown in **Table 3**.

**Table 3:** Comparison of AUCs among models.

Cohorts	Model	Rad-score 1	Rad-score 2	Rad-score 3	MRI- LN
Training	Rad-score 1	/	0.553	0.300	0.062
	Rad-score 2	0.553	/	0.654	0.011
	Rad-score 3	0.300	0.654	/	0.001
	MRI- LN	0.062	0.011	0.001	/
Validation	Rad-score 1	/	0.672	0.571	0.257
	Rad-score 2	0.672	/	0.814	0.127
	Rad-score 3	0.571	0.814	/	0.037
	MRI- LN	0.257	0.127	0.037	/

MR-LN: MRI-reported LNM status.

### Radiomics Nomogram Construction and Evaluation

**Figure 4:**
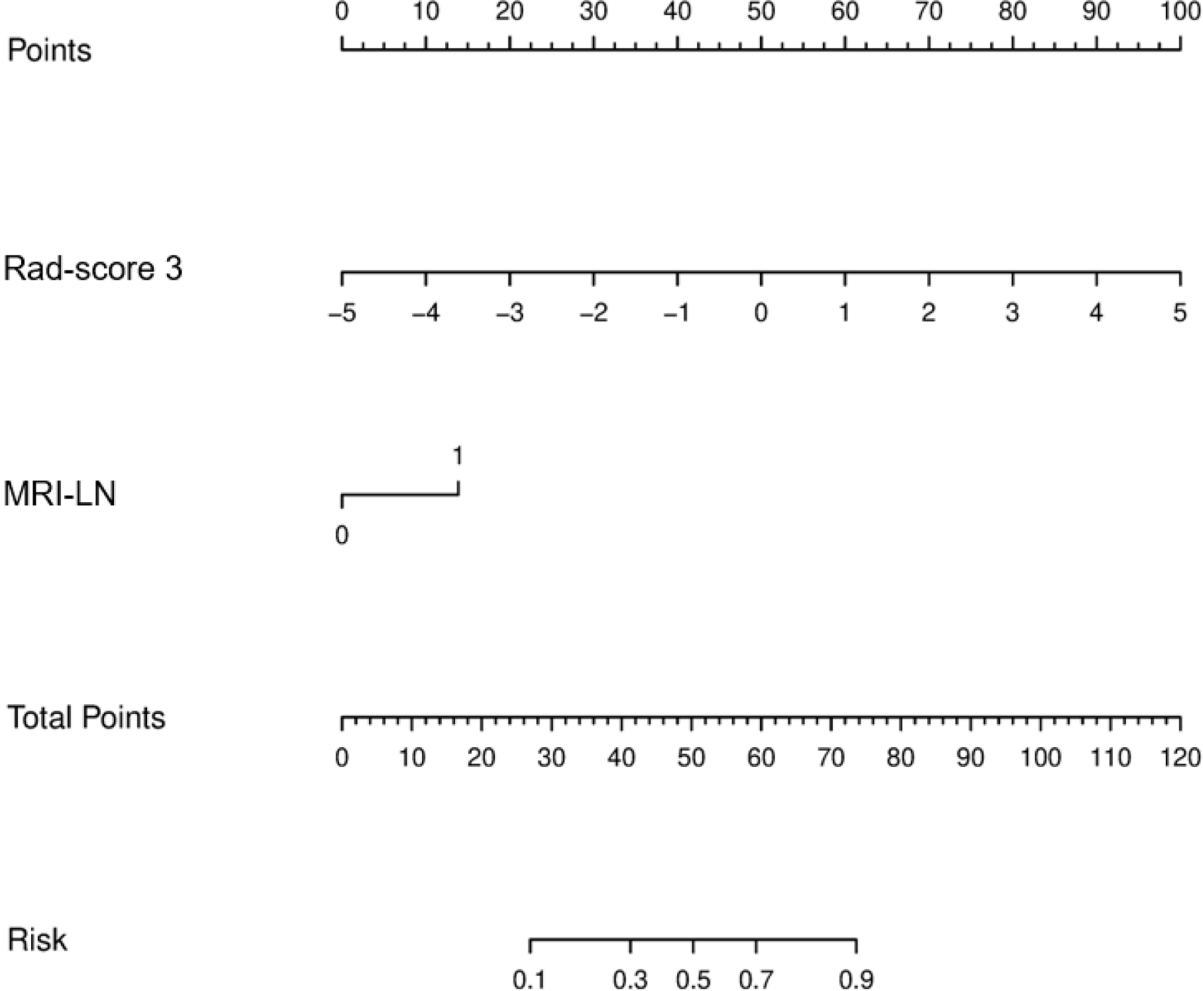
Radiomics nomogram incorporating the MRI-reported LN status and rad-score 3. MRI-LN, MRI-reported LN status.

**Figure 5:**
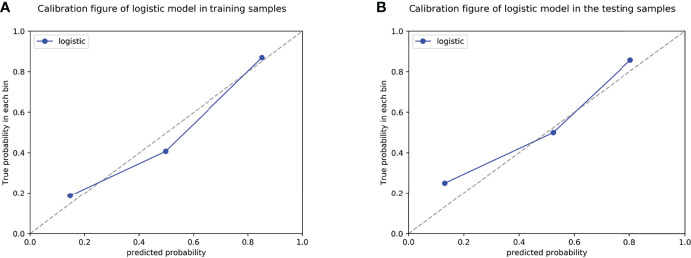
Calibration curves of the radiomics nomogram in the training cohort **(A)** and validation cohort **(B)**.

**Figure 6:**
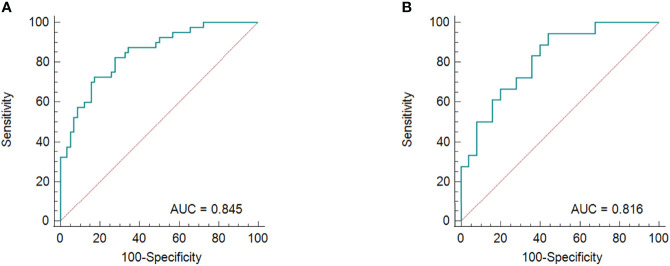
The ROC curves for the radiomics nomogram in the training group **(A)** and validation cohort **(B)**.

**Figure 7:**
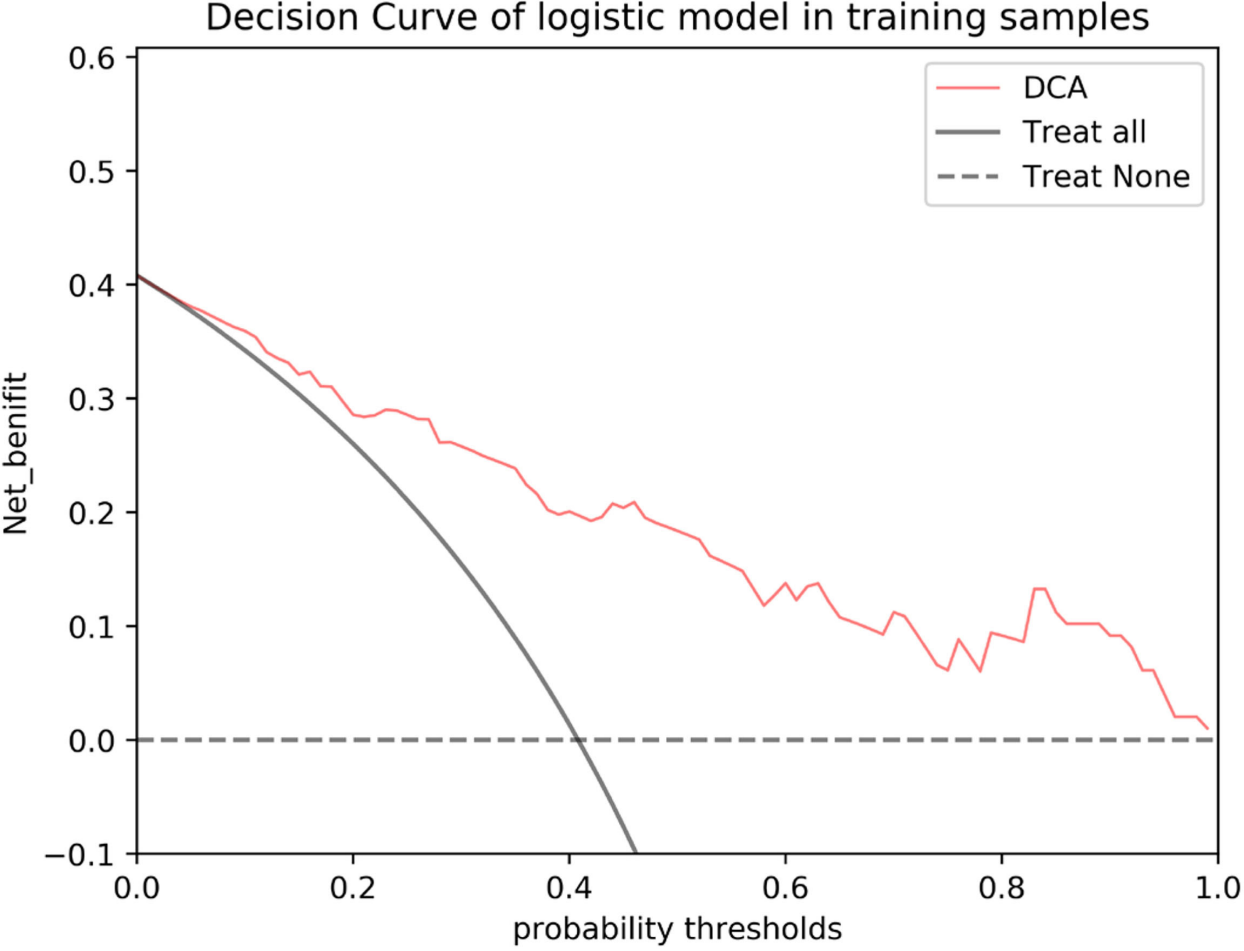
DCA of the nomogram based on MRI-reported LN status and rad-score 3 in the validation cohort. The red line, gray line, and horizontal dotted line represent the net benefit of the nomogram, treat-all strategy, and treat-none strategy, respectively.

The results of the univariate and multivariate logistic regression analyses are presented in **Table 4**. Univariate analysis revealed significant differences in the MRI-reported lymph node status and rad-score 3 between LNM and nLNM patients in the training cohort, and they were identified as independent predictors of LNM by multivariate logistic regression analysis. The radiomics nomogram constructed by incorporating independent predictors is shown in **Figure 4**. The Hosmer–Lemeshow test showed good calibration of the nomogram in both the training and validation cohorts (*P*=0.938 and 0.924), and the calibration curves exhibited good calibration ability (**Figure 5**). The AUC values of the nomogram for predicting LNM of PDAC in the training and validation cohorts were 0.845 [95% confidence interval (CI), 0.777-0.907] and 0.816 (95% CI, 0.698-0.914), with AUCs of 0.828 and 0.680 for specificity and AUCs of 0.700 and 0.722 for sensitivity, respectively. ROC curves are shown in **Figure 6**. The DCA results for the validation cohort are shown in **Figure 7**. We found that the nomogram can obtain better net benefits than the “treat-all” or “treat-none” strategies under a wide probability threshold.

**Table 4:** Univariate and multivariate logistic regression analyses of the clinical parameters and rad-scores.

Characteristic	Univariate analysis		*P*	Multivariate analysis		*P*
	OR	95% CI		OR	95% CI	
Age	0.991	0.955-1.029	0.651			
Sex	0.833	0.414-1.676	0.608			
Location	0.610	0.307-1.213	0.159			
Size	1.090	0.875-1.357	0.441			
CA19-9	1.000	1.000-1.000	0.874			
CA125	0.998	0.992-1.004	0.509			
CEA	1.001	0.999-1.003	0.535			
mTs	1.115	0.552-2.251	0.762			
MRI-reported LN status	5.153	2.219-11.966	0.000	4.251	1.309-13.808	0.016
Rad-score 3	2.471	1.756-3.477	0.000	2.448	1.571-3.814	0.000

mTs, MRI tumor stage.

## Discussion

PDAC is a gastrointestinal tumor with extremely high malignancy and poor prognosis, which is largely attributed to difficulties in early diagnosis and the limited number of treatment options available for this disease (1). Lymph node status is the key factor in developing appropriate treatment strategies and improving the prognosis of patients (30, 31). However, traditional MRI can only make a preoperative diagnosis of LNM according to the lymph node size, morphology and signal characteristics, which can be subjective, leading to low diagnostic sensitivity (11, 12, 32). In our study, we obtained a sensitivity of 40%, similar to the literature. Moreover, logistic regression analysis showed that MRI-reported lymph node status was the only independent risk factor among all the clinical characteristics analyzed. A previous study reported that CT-reported lymph node status was the only independent risk factor, but it had relatively low predictive efficacy (AUC=0.63) (33). Although the CA19-9 level may predict the prognosis of patients with pancreatic cancer (20, 34, 35), it could not be confirmed as a risk factor for preoperative LNM in our study, which warrants further investigation.

Radiomics is an advanced method for quantitative analysis that can reveal information from microscopic features that are not easily observable by the naked eye in medical imaging (17, 36). In recent years, various studies have attempted to predict LNM based on MRI radiomic analysis of primary lesions (22, 37–43). To the best of our knowledge, only one study has analyzed the predictive efficacy of radiomics based on MRI for LNM of PDAC (43), but only the arterial phase of the T1WI enhanced sequence was used. T2WI can reflect the signal intensity of the tumor tissue and its structure, and the enhanced sequence can better reflect tumor-related information such as internal heterogeneity and vascular regeneration (18–20, 24, 44). Based on the two sequences and by incorporating the independent predictor of MRI-reported lymph node status, we constructed a model with good predictive efficacy, with an AUC of 0.845 in the training cohort. This result is similar to findings reported in previous studies on multiparametric MRI-based radiomics nomograms for predicting LNM of lung adenocarcinoma, bladder cancer, and cervical cancer, with AUCs ranging from 0.820 to 0.856 in the training cohort (37–39).

We delineated a 3D ROI containing comprehensive information (45), and 1037 features were extracted, including high-order features. After dimensionality reduction, we found that rad-score 1 and rad-score 2 mainly consisted of wavelet features (7/8, 5/6), and rad-score 3 consisted only of wavelet features, indicating that wavelet features better reflect the biological characteristics and heterogeneity of tumors. Wavelet filters can help to sharpen the image and eliminate noise (46), and the features of wavelet filters can represent the signal intensity distribution or grayscale distribution in the tissue (47). For example, among the first-order features, “LLH_firstorder_Minimum” and “LLH_firstorder_Mean” describe the minimum and mean gray intensity of the tumor region, respectively, and the difference in the grayscale intensity distribution is shown by “LLL_firstorder_Kurtosis”. In addition, “LLH_glszm_SizeZoneNonUniformity” and “HHH_gldm_DependenceNonUniformityNormalized” represent the heterogeneity of the tumor tissue. “HHH_glszm_SmallAreaEmphasis” is expressed as a greater value with smaller size zones and more fine textures. This study confirmed that “MaximumProbability”, “MaximumProbability” and “MaximumProbability” were the most meaningful among all the GLCM features, showing differences in the regional signal intensity distribution, gray level skewness, uniformity and linear dependency within PDAC tissues with LNM or nLNM tendency (47). The value of some of the wavelet features we obtained has also been confirmed in recent studies on LNM of rectal cancer and cervical cancer, especially the features based on T2WI (21, 24, 48). We also found that only six features from T2WI were retained—fewer than those retained from PVP (eight features). However, the former had a larger AUC score, although the difference was not statistically significant. This may be related to the higher tissue resolution provided by T2WI and greater influence of upper abdominal respiratory artifacts in dynamic enhanced scanning, which requires further studies with histopathology.

There were several limitations to this study. First, the sample size was small, so selection bias may exist. Because this was a single-center study, the application of the radiomics nomogram was limited as well; more data from multicenter and multiple MRI scanners are needed to verify the accuracy and stability of our radiomics model. Second, volume effects and respiratory motion artifacts could not be completely avoided when delineating the tumor boundaries. Third, only the portal vein phase of the multiphase enhancement sequences was analyzed, analyses and comparisons of each phase could be performed to determine their predictive value in future work, DWI sequence could be studied as well.

In conclusion, the results of our study demonstrated that a radiomics nomogram based on dual-parametric MRI imaging could successfully predict LNM and nLNM of PDAC. This method shows higher specificity and sensitivity than traditional MRI can provide, allowing clinicians to be able to prepare more thoroughly before performing surgical procedures. In addition, the use of this nomogram can ultimately improve the prognosis of patients.

## Data Availability Statement

The original contributions presented in the study are included in the article/supplementary material. Further inquiries can be directed to the corresponding author.

## Ethics Statement

The studies involving human participants were reviewed and approved by Zhejiang Provincial People’s Hospital, Affiliated People’s Hospital, Hangzhou Medical College. Written informed consent for participation was not required for this study in accordance with the national legislation and the institutional requirements.

## Author Contributions

JC designed the study and revised the final manuscript. LS and LW analyzed the data and wrote the first draft. GW auxiliary analyzed the data. CW and YZ performed the image acquisition and analyzed the data. All authors contributed to the article and approved the submitted version.

## Funding

This research was supported by Zhejiang Provincial Natural Science Foundation of China (No. LGC22H180002) and Zhejiang Medical and Health Science and Technology Project (No. 2020KY406, 2021KY508).

## Conflict of Interest

Author YW was employed by General Electric Healthcare.

The remaining authors declare that the research was conducted in the absence of any commercial or financial relationships that could be construed as a potential conflict of interest.

## Publisher’s Note

All claims expressed in this article are solely those of the authors and do not necessarily represent those of their affiliated organizations, or those of the publisher, the editors and the reviewers. Any product that may be evaluated in this article, or claim that may be made by its manufacturer, is not guaranteed or endorsed by the publisher.
